# No safe renal warm ischemia time—The molecular network characteristics and pathological features of mild to severe ischemia reperfusion kidney injury

**DOI:** 10.3389/fmolb.2022.1006917

**Published:** 2022-11-16

**Authors:** Ya-Lei Chen, Huai-Kang Li, Lei Wang, Jian-Wen Chen, Xin Ma

**Affiliations:** ^1^ Department of Critical Care Medicine, Capital Medical University Electric Power Teaching Hospital/State Grid Beijing Electric Power Hospital, Beijing, China; ^2^ Senior Department of Urology, The Third Medical Centre of PLA General Hospital, Beijing, China; ^3^ Department of Nephrology, State Key Laboratory of Kidney Diseases, Beijing Key Laboratory of Kidney Disease Research, First Medical Center of Chinese PLA General Hospital, Nephrology Institute of the Chinese People’s Liberation Army, National Clinical Research Center for Kidney Diseases, Beijing, China

**Keywords:** acute kidney injury, pathological features, ischemia reperfusion kidney injury, molecular network characteristics, mild to severe ischemia reperfusion injury

## Abstract

Ischemic acute kidney injury (AKI) has always been a hot and difficult research topic in the field of renal diseases. This study aims to illustrate the safe warm ischemia time of kidney and the molecular network characteristics and pathological features of mild to severe ischemia reperfusion kidney injury. We established varying degrees of renal injury due to different ischemia time (0 min, 16 min, 18 min, 20 min, 22 min, 24 min, 26 min, 28 min, and 30 min) on unilateral (left kidney) ischemia-reperfusion injury and contralateral (right kidney) resection (uIRIx) mouse model. Mice were sacrificed 24 h after uIRIx, blood samples were harvested to detect serum creatinine (Scr), and kidney tissue samples were harvested to perform Periodic Acid-Schiff (PAS) staining and RNA-Seq. Differentially expressed genes (DEGs) were identificated, time-dependent gene expression patterns and functional enrichment analysis were further performed. Finally, qPCR was performed to validated RNA-Seq results. Our results indicated that there was no absolute safe renal warm ischemia time, and every minute of ischemia increases kidney damage. Warm ischemia 26min or above in mice makes severe kidney injury, renal pathology and SCr were both significantly changed. Warm ischemia between 18 and 26 min makes mild kidney injury, with changes in pathology and renal molecular expression, while SCr did not change. No obvious pathological changes but significant differences in molecular expression were found less than 16min warm ischemia. There are two key time intervals in the process of renal ischemia injury, 0 min–16 min (short-term) and 26 min–28 min (long-term). Gene expression of immune-related pathways were most significantly down-regulated in short-term ischemia, while metabolism-related pathways were the mainly enriched pathway in long-term ischemia. Taken together, this study provides novel insights into safe renal artery occlusion time in partial nephrectomy, and is of great value for elucidating molecular network characteristics and pathological features of mild to severe ischemia reperfusion kidney injury, and key genes related to metabolism and immune found in this study also provide potential diagnostic and therapeutic biomarkers for AKI.

## 1 Introduction

Acute kidney injury (AKI) is a multidisciplinary disease, at present, the basic research work of AKI is mainly focused on the fields of nephrology, intensive care unit and kidney transplantation (Bellomo et al., 2012; Zhang et al., 2020). In these fields, bilateral or isolated renal ischemia is commonly faced, and the incidence of AKI is very high. For example, the incidence of hospital-acquired AKI is approximately up to 20% in the intensive care unit (Gonsalez et al., 2019). Due to the limited treatment of AKI, there are still a high proportion of patients with AKI who progress to chronic kidney disease (CKD) and end-stage renal disease, which need kidney dialysis or waiting for kidney transplantation (Chen et al., 2020; Uber and Sutherland, 2020). Despite extensive experimental and clinical investigations, the underlying pathogenesis and progression mechanisms of AKI have not been fully clarified.

Ischemia-reperfusion is one of the main cause of AKI (Melo Ferreira et al., 2021). In urology, the renal artery needs to be clipped during nephron sparing surgery or partial nephrectomy (Weight et al., 2010; Soranno et al., 2019). Transient occlusion of the renal artery can control surgical blood loss, facilitate tumor resection, and wound suture. However, the occlusion of the renal artery also causes transient renal ischemia and potential renal injury (Thompson et al., 2010). At present, 25 min of renal warm ischemia is generally recognized as the threshold for AKI (Funahashi et al., 2009). Other studies have shown that warm ischemia time of laparoscopic partial nephrectomy over 20 min is correlated with poor preoperative and postoperative glomerular filtration rate, and the incidence of postoperative complications increases 2.54 times for each extension of warm ischemia time of laparoscopic partial nephrectomy for 9 min (Lane et al., 2008). Based on the analysis of 362 kidney patients undergoing partial nephrectomy, Thompson et al. of the Department of Urology at Mayo Clinic found that a longer warm ischemia time was associated with both short-term and long-term renal complications, suggested that every minute of renal artery occlusion during surgery was important (Thompson et al., 2010). These academic debates indicate that the safe time of warm ischemia is still controversial, and the specific relationship between renal ischemia time and AKI requires a larger clinical sample size and clear basic research evidence.

The experimental mouse models of renal ischemia/reperfusion injury (IRI) have been widely applied to study the pathogenesis and injury outcome of ischemic AKI (Chen et al., 2020). IRI mouse model can well simulate renal injury caused by renal artery occlusion in human partial nephrectomy. In this study, we established a relatively stable IRI model with unilateral ischemia (left kidney) and contralateral (right kidney) resection (uIRIx) to explore the changes of injury, molecular network characteristics and pathological features of mouse kidney with different degrees of ischemia. We systematically examined the renal pathology at different ischemia times, and identified the molecular characteristics of gene expression associated with different ischemia times by performing RNA sequencing (RNAseq). Our study provides basic research evidence for the effect of ischemic time on renal damage in partial nephrectomy of urology, and offers resources for clinical and research communities, and brings basis and new ideas for AKI research.

## 2 Materials and methods

### 2.1 Animal model construction and detection

Wild-type C57BL/6 mice (18–22 g) were purchased from the Animal Center of Chinese PLA General Hospital. These mice were housed in a specific pathogen-free facility under a 12 h light/12 h dark cycle with free access to food and water. Mice were randomly assigned to several groups based on different ischemia time: 0 min, 16 min, 18 min, 20 min, 22 min, 24 min, 26 min, 28 min, and 30 min. To induce unilateral ischemia reperfusion injury and the contralateral kidney resection AKI (uIRIx), mice were placed on 37°C warm plate (Yuyan instruments, Beijing, China) to keep the temperature of kidney all through the surgery; the right kidney was excised, and left kidney pedicle was clipped for indicated time using microaneurysm clamp. Mice were then sacrificed 24 h after uIRIx, blood and kidney tissue samples were harvested for further processing. The animal protocol and all animal procedures were reviewed and approved by the Institutional Animal Care and Use Committee (IACUC) of the Chinese PLA General Hospital, and the permit number is 2019-X15-97.

### 2.2 Blood and histopathological assessment of kidney injury

Blood samples were collected from the vena cava at the indicated time points, and the serum was separated by centrifugation at 3,000 rpm for 15 min at 4°C and then sent to the PLA General Hospital Biochemistry Department to detect serum creatinine (SCr). A quarter of the left kidney was fixed in 4% formaldehyde, dehydrated, and embedded in paraffin. Histopathological examination was performed by Periodic Acid-Schiff (PAS) staining of kidney tissue section (2 μm) to evaluate tubular injury.

### 2.3 High-throughput RNA sequencing (RNA-Seq) and preprocessing

In order to explore the molecular characteristics of kidney injury at different ischemic times, the left ischemia kidney in each mouse was collected and the RNA was extracted to perform RNA-Seq (Beijing Genomics Institute, Beijing, China). All raw RNA-Seq data were uploaded to the GEO database GSE192883 (https://www.ncbi.nlm.nih.gov/geo/query/acc.cgi?acc=GSE192883). The preprocessing of the gene expression profile data, which includes the background correction, the quantile normalization, the median polish summarization, and the log2 transformation, was performed by R software (https://www.r-project.org/) and RStudio software (https://www.rstudio.com/) as we described before (Chen et al., 2020). Principal component analysis (PCA) was performed by scatterplot3d package and princomp function in R software.

### 2.4 Identification of differentially expressed genes

The linear model for high-throughput data analysis (limma) in Bioconductor (http://www.bioconductor.org/) was applied to find DEGs by comparing expression value between different ischemia groups. Differential expression was calculated using an empirical Bayes model. The criteria for the statistically significant difference of DEGs was | log2 fold change (FC)| > 1 in expression and adjusted *p*-value (false discovery rate, FDR) < 0.05. The overlapping DEGs were further analyzed by UpSetR package in R software. We obtained a union DEGs set, which included the combination of DEGs obtained in each ischemia group compared with the ischemia 0min and 16min groups.

### 2.5 Time-dependent gene expression patterns profiling

We applied the fuzzy c-means algorithm in the Mfuzz R package (Kumar and Futschik, 2007) to perform noise-robust soft clustering analysis and visualize the time-dependent expression patterns of the genes in union DEGs set. Soft clustering is more accurate than hard clustering with more robustness to noise and less information loss, and Mfuzz R package is one of the software packages for soft clustering (Kumar and Futschik, 2007; Yang et al., 2021). The average RPKM value for each gene at each time point was used as the input. After standardization, genes in union DEGs set were assigned to 8 clusters, such as continuous increase, continuous decrease, increase first then decrease, and decrease first then increase etc., according to the changes in the gene expression with ischemia time.

### 2.6 Functional enrichment analysis

Gene ontology (GO, http://www.geneontology.org) analysis has been widely used to annotate the gene function. The categories of GO include the cellular component (CC), the biological process (BP), and the molecular function (MF) terms (Chen et al., 2020). Kyoto Encyclopedia of Genes and Genomes (KEGG, http://www.genome.jp/kegg) is a database containing the information of genes related to metabolic and regulatory pathways (Yang et al., 2021). In the present study, the clusterProfiler R package (Yu et al., 2012) was used to conduct the GO and KEGG functional analysis for genes enriched in each cluster, respectively. The *p*-value of enriched pathways was calculated by a hyper geometric distribution test. Subsequently, the *p*-value was revised using BH (Benjamini and Hochberg) method, and the adjusted *p*-value < 0.05 was served as the cut-off criterion for selecting significant enrichment pathways. We also used clusterProfiler R package with the same threshold value, as mentioned above, to conduct the gene set enrichment analysis (GSEA) analyses for genes compared between 16 min and 0 min, between 28 min and 26 min, respectively. The criteria for the statistically significant difference of GSEA was |normalized enrichment score (NES)| >0.4 and adjusted *p*-value < 0.05.

### 2.7 PPI network construction and analysis of modules

Considering that proteins rarely work alone, it is necessary to study the interactions among proteins. The Search Tool for the Retrieval of Interacting Genes/Proteins (STRING, http://string-db.org/) is an online biological resource database that is commonly used to identify the interactions between known and predicted proteins (Szklarczyk et al., 2015). By searching the STRING database, the PPI network of the genes in each cluster acquired by soft clustering above were selected with a score>0.4, and the PPI network was visualized by Cytoscape software (http://www.cytoscape.org/). In the PPI network, each node stands for a gene or a protein, and edges represent the interactions between the nodes. We then used the plug-in named Molecular Complex Detection (MCODE) by Cytoscape to filter the modules of the PPI network. The first module of each cluster was selected.

### 2.8 Gene expressions by quantitative real-time PCR

The frozen tissue samples were lysed in TRIzol reagent (Invitrogen, Carlsbad, CA) and total RNA was extracted according to the manufacturer’s instructions. The levels of transcripts were determined by qPCR using TransStart^®^ Top Green qPCR SuperMix (AQ131, Transgen, Beijing, China) on an Applied Biosystems 7,500 system PCR cycler (Applied Biosystems, Foster City, CA, United States). The data were normalized to 18s expression. Primers were obtained from Beijing Genomics Institute (Beijing, China). The list of primers was presented in Supplementary Table S1.

### 2.9 Statistical analysis

The statistical analyses used are specified in the figure legends.

## 3 Results

### 3.1 Pathological features of kidney injury at different ischemia time pionts

**FIGURE 1:**
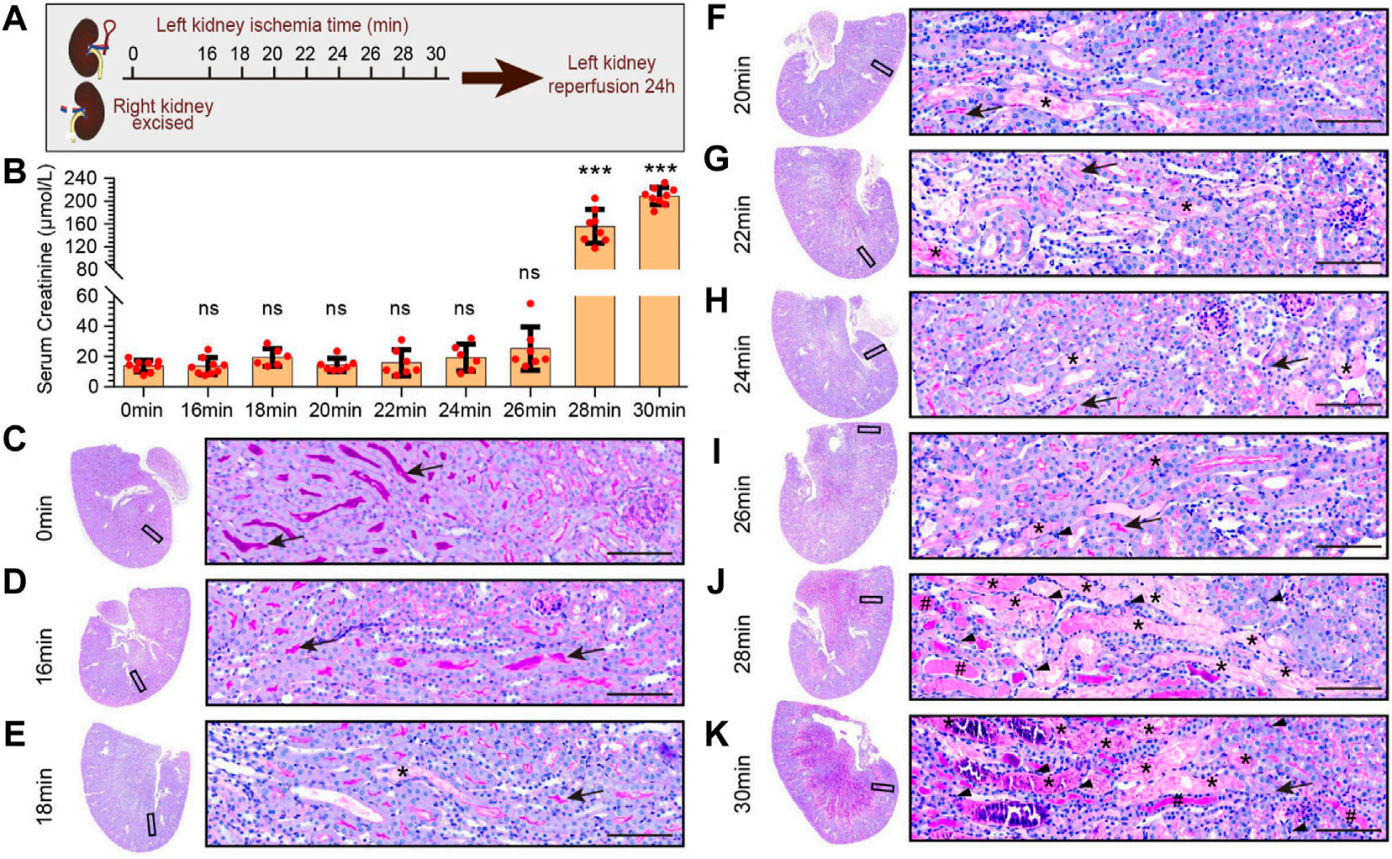
Pathological features of kidney injury at different ischemia times. **(A)** The experimental design of the unilateral ischemia reperfusion injury and the contralateral kidney resection AKI (uIRIx) model. Mice were randomly assigned to 9 ischemia groups based on different ischemia time of left kidney: 0 min, 16 min, 18 min, 20 min, 22 min, 24 min, 26 min, 28 min, and 30 min, and the mice were sacrificed 24 h after reperfusion. **(B)** The serum creatinine levels in different ischemia groups 24 h after reperfusion. Statistical analysis: firstly, we performed one-way ANOVA analysis on the 9 groups, and found statistically significant differences in mean values among the samples; And then, Dunnett-t test was used to make multiple comparisons of mean differences between each ischemia group and sham group. n = 6–9 mice per group, ns indicates no significant, ***p < 0.001, compared to 0min group **(C–K)** Representative micrographs of PAS staining showed the pathological features of kidney injury at different ischemia times: 0min group **(C)**, 16 min group **(D)**, 18 min group **(E)**, 20 min group **(F)**, 22 min group **(G)**, 24 min group **(H)**, 26 min group **(I)**, 28 min group **(J)**, and 30 min group **(K)**. Scale bars: 100 μm. The arrow showed the brush border of normal tubule, the asterisk (*) showed the blocked tubule, the triangle showed the infiltrated inflammatory cell, and the well sign (#) showed the protein tubule.

To explore the pathological characteristics of kidney injury at different ischemia times, we adopted the uIRIx model, which were more stable than bilateral ischemia reperfusion kidney injury AKI model. Mice were randomly assigned to 9 ischemia groups based on different ischemia times of left kidney: 0 min, 16 min, 18 min, 20 min, 22 min, 24 min, 26 min, 28 min, and 30 min (Figure 1A), ischemia 0min was the control group. As shown in Figure 1B, after reperfusion for 24 h, the serum creatinine of 28 min and 30 min groups were significantly higher than that of 0 min group (*p* < 0.05), while there was no statistical difference among 16 min, 18 min, 20 min, 22 min, 24 min, or 26 min with 0 min group. The serum creatinine dramatically changed between 26 min and 28 min of ischemia at the condition of 37°C.

PAS staining of kidney (Figures 1C–K) showed that the renal cortex and medulla structures of mice in the 0min and 16min groups were normal, with no ablation of the brush edge of renal tubules, no dilation of renal tubules and no tubular formation. In ischemia 18min group, there was scattered cellular cast in the cortical and medulla area. In 20min, 22min, and 24min groups, the cellular casts in the cortex and medulla area increased gradually, and in 26min group, there was small patches of renal tubular obstruction. In ischemia 28min group, there was necrosis, tubule blockage formed by exfoliation of large patchy renal tubular epithelial cells, and inflammatory cell infiltration in the interstitium, as well as patchy protein tube type. In ischemia 30min group, almost all areas were filled with tubule cast, protein cast, and inflammatory cell infiltration.

### 3.2 Expression characteristics of injury-related molecules of kidney injury at different ischemia time points

**FIGURE 2:**
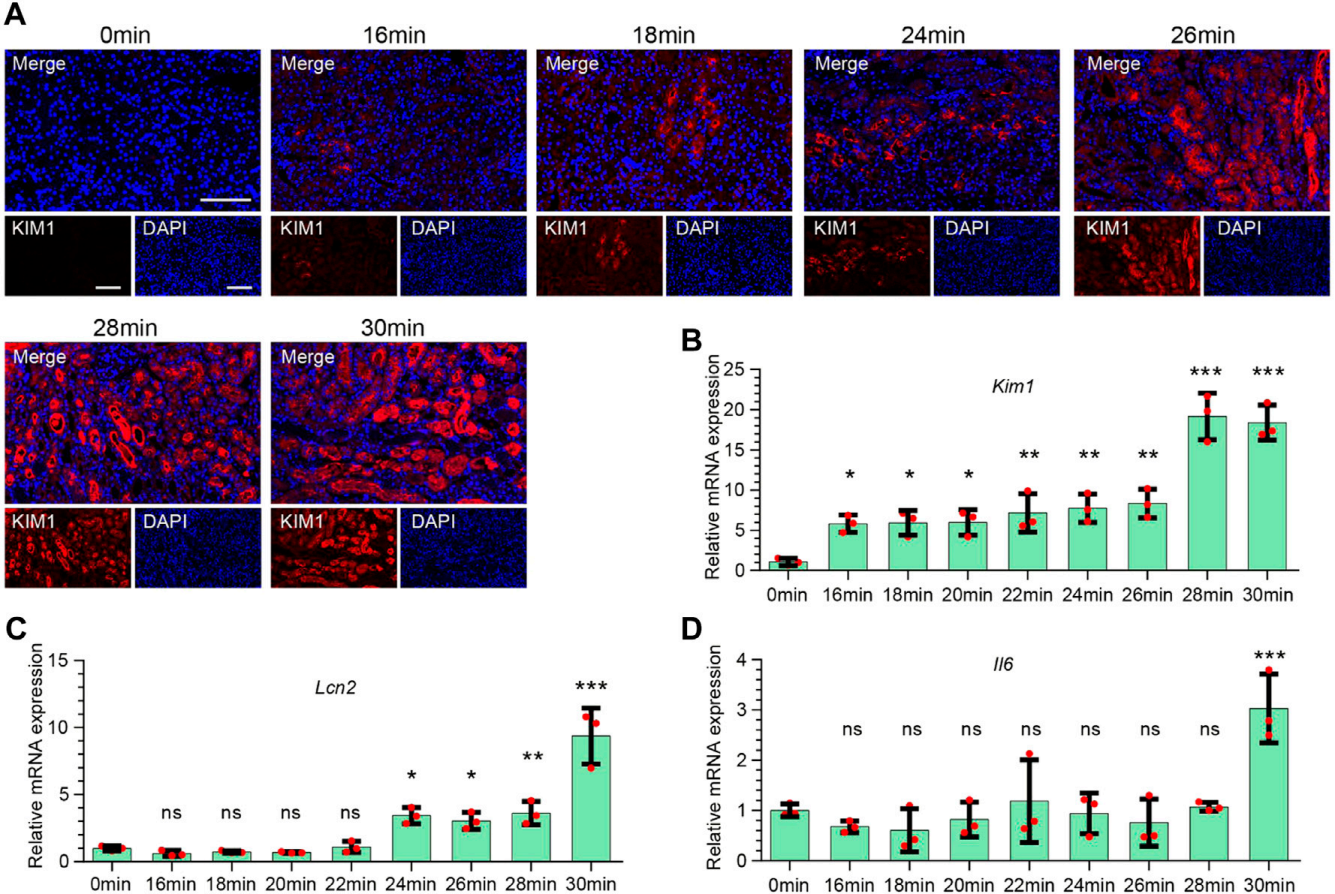
Expression characteristics of injury-related molecules of kidney injury at different ischemia time points. **(A)** Representative immunofluorescence staining of KIM1 expression in renal tissues at different ischemia time points. Red stands for KIM1. Scale bars: 100 μm **(B–D)** The mRNA expression of Kim1 **(B)**, Lcn2 **(C)**, and Il6 **(D)** in renal tissues at different ischemia time points. One-way ANOVA and Dunnett-t test, n = 3 per group, ns indicates no significant, *p < 0.05, **p < 0.01, ***p < 0.001, compared to 0min group.

To further elucidate the expression of injury-related molecules at different renal ischemia time points, we performed immunofluorescence staining on KIM1 (Figure 2A). The results found that the expression of KIM1 began at 16 min of ischemia, and then increased with the increase of ischemia time. At the same time, we performed qPCR to detect the expression of Kim1 (Figure 2B), Lcn2 (Figure 2C), and Il6 (Figure 2D), and found that Kim1 was highly expressed at 16 min and continued to increase at 30 min after ischemia. The expression of Lcn2 (Ngal) began to increase in the 22 min ischemia group, and gradually increased with the prolongation of ischemia time. However, the expression of Il6 was significantly increased at 30 min of ischemia, with no significant in less than ischemia 28 min.

### 3.3 High-throughput RNA sequencing and identification of differentially expressed genes between groups

**FIGURE 3:**
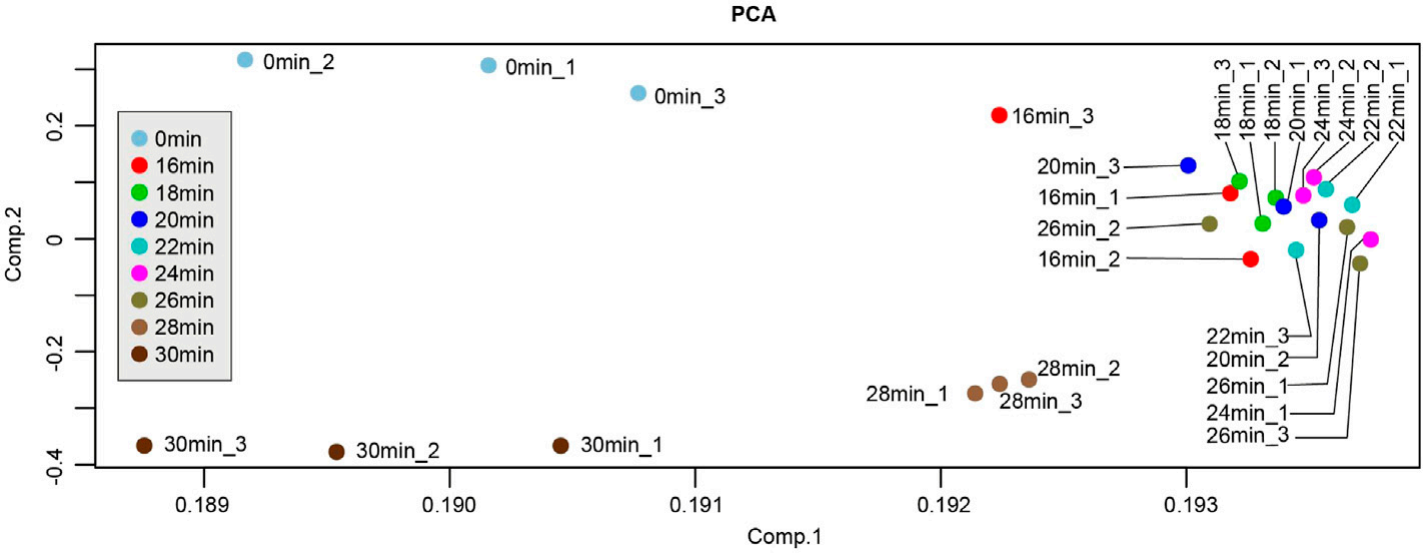
Principle component analysis (PCA) plot of samples from different ischemia times. Each point represents a sample. PCA plot showed the tight clustering of biological replicates and distinct clustering between groups.

In order to explore the molecular characteristics of kidney injury at above different ischemic times, triplicate left ischemia kidneys in every group were collected to perform RNA-Seq. Before the next analysis, we performed the quality control for every raw expression data to guarantee the quality. The normalized box diagram showed that the data was well preprocessed after normalization. Next, we applied PCA analysis to those 27 samples (different colors) to visualize sample clusters. The results showed that samples at the same time points were clustered (Figure 3), indicating good correlation between the biological repeats. Notably, samples of 16 min, 18 min, 20 min, 22 min, 24 min, and 26 min localized close to each another, suggesting that those time points have similarities in transcriptome.

**FIGURE 4:**
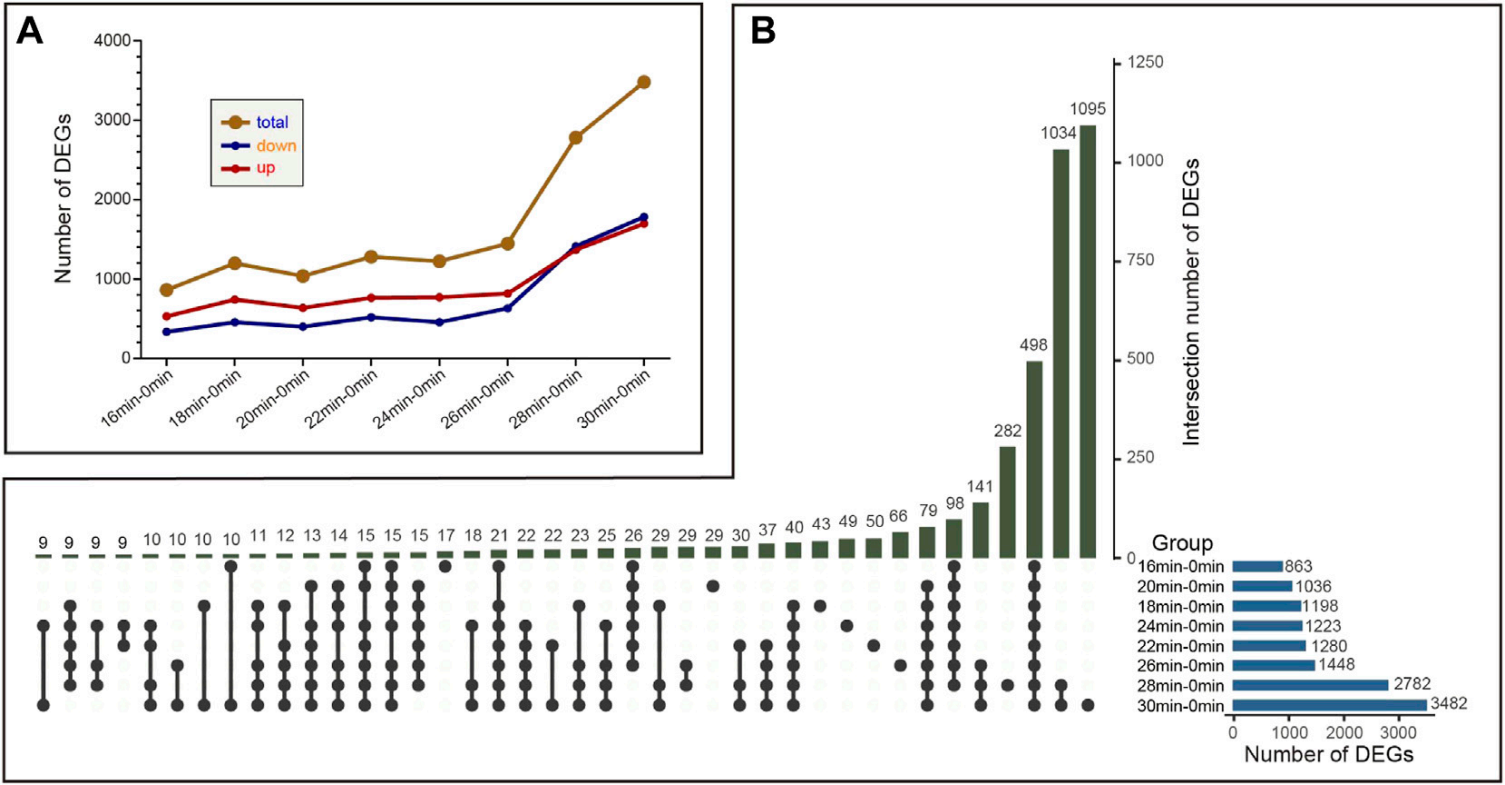
The variation trend of the number of differentially expressed genes (DEGs) between 0 min group and different ischemia groups. **(A)** The variation trend of the number of DEGs among different compared groups **(B)** The number of intersected DEGs between different compared groups. The number above stands for the number of DEGs that intersect between different groups.

DEG analysis was performed in all samples using cutoff values of a fold change≥2 and an adjusted *p*-value < 0.05. With the increase of ischemia time from 16 min to 26 min, the number of DEGs was gradually but slowly increased, from 863 to 1,448. While in the group of 28 min vs 0 min, the number of DEGs suddenly increased at 2,782. The most pronounced DEGs were in the group of 30 min vs 0 min, with 1,698 upregulated and 1784 downregulated genes (Figure 4A). Overall, the kidney transcriptome was similar at different time points of the 16 min, 18 min, 20 min, 22 min, 24 min, and 26 min. These findings are consistent with PCA results.

The overlapping DEGs were further analyzed by UpSetR package in R software. The number of unique DEGs that only found in 16 min–0 min group, 18 min–0 min group, 20 min–0 min group, 22 min–0 min group, 24 min–0 min group, 26 min–0 min group, 28 min–0 min group, and 30 min–0 min group were 17,43,29,50,49,66,1034,1,095, respectively (Figure 4B). Moreover, 498 genes were differentially expressed in each group. We also obtained a union DEGs set, which included the combination of DEGs obtained in each ischemia group compared with the ischemia 0 min and 16 min groups. This union DEGs set contains 4,519 genes.

### 3.4 The time-dependent gene expression patterns of genes in union DEGs set

**FIGURE 5:**
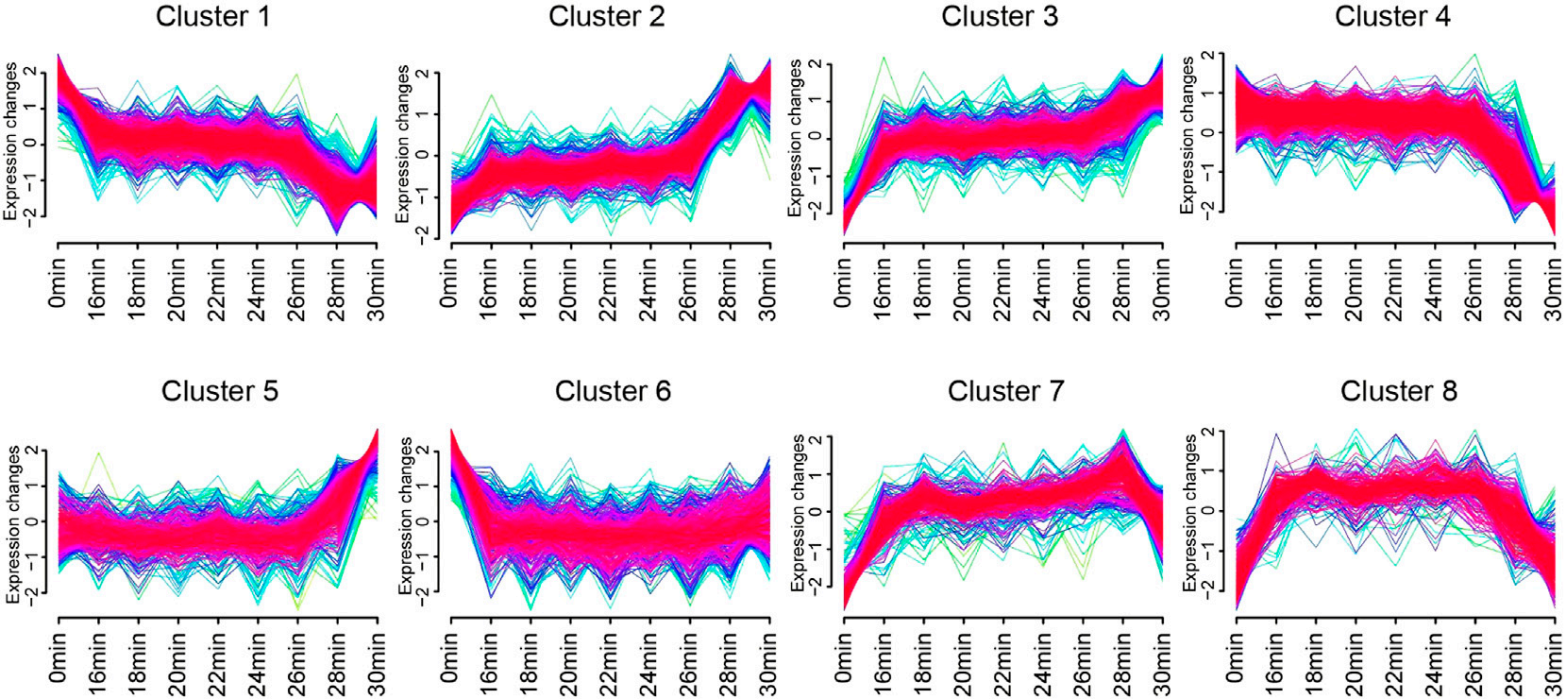
The time-dependent gene expression patterns of genes in union DEGs set. Eight clusters identified based on gene expression of genes in union DEGs set by Mfuzz R package. Curves in yellow or green denote genes with small membership values, while curves in red or purple correspond to genes with big membership values.

In order to visualize the expression patterns and characteristics of all 4,519 union DEGs, we applied the Mfuzz R package on them. After standardization, genes in union DEGs set were assigned to 8 clusters (Figure 5). The expressions of genes in Cluster 1 were highest in 0 min group, declined to a plateau in 16 min group to 26 min group, and reached the lowest in 28 min group and 30 min group. The expressions of genes in cluster 2 were opposite with cluster 1. The expressions of genes in Cluster 3 were lowest in 0 min group, increased to a plateau in 16 min group to 26 min group, increased then in 28 min group, and reached the highest in 30 min group. The difference between cluster 2 and cluster 3 is that the expressions of genes in Cluster 3 were much higher in 30 min group than 28 min group. The expressions of genes in Cluster 4 were low in 0min group to 26 min group, and continuous decrease in 28 min group and 30 min group, the expressions of genes in cluster 5 were opposite with cluster 4. The expressions of genes in Cluster 6 were highest in 0 min group, and decreased to a plateau in 16 min group to 30 min group. The expressions of genes in cluster 7 and cluster 8 were firstly increased and then decreased after reaching a plateau. The difference between these two clusters was that the expressions of genes of in cluster 7 start to decreased in the 30 min group, while those of cluster 8 began to decline in the 28 min group.

By searching the STRING database, the PPI network of the genes in each cluster acquired by soft clustering above were selected with a score>0.4. We then used the plug-in named Molecular Complex Detection (MCODE) by Cytoscape to filter the modules of the PPI networks. The first module of each cluster was selected (Supplementary Figure S1).

### 3.5 GO and KEGG pathway enrichment analyses of genes in each cluster

To gain insight into the biological roles of each cluster, KEGG pathway and GO term analyses were performed. The results showed that genes in each cluster were associated with distinct biological processes. Genes in Cluster 1, cluster 4 and cluster 6 were down-regulated with the prolongation of ischemia time. Cluster 1 was mainly associated with metabolic-related processes, such as “fatty acid metabolic process”, “purine-containing compound metabolic process”, “valine, leucine and isoleucine degradation”, “pyruvate metabolism”, and “tryptophan metabolism”. In addition, immune-related processes such as “complement and coagulation cascades”, “antigen processing and presentation”, and “Th1 and Th2 cell differentiation” were also enriched in Cluster 1. Similar to Cluster 1, Cluster 4 genes were also mainly involved in metabolic-related processes.

Genes in Cluster 6 were mainly involved in immune-related processes, such as “antigen processing and presentation”, “cytokine-cytokine receptor interaction”, “response to interferon-gamma”, “leukocyte mediated cytotoxicity”, and “T cell mediated cytotoxicity” (Supplementary Figure S2). These results indicated that, the main event of decline expression genes from ischemia 0 min to ischemia 16 min were immune-related processes, while genes with sharply decreased expression from ischemia 26 min to ischemia 28 min were mainly enriched in metabolism-related pathways.

Genes in Cluster 2, cluster 3 and cluster 5 were up-regulated with the prolongation of ischemia time. Cluster 2 were mainly involved in cell junction-related processes, such as “tight junction”, “focal adhesion”, “cell junction assembly”, and “cell-matrix adhesion”. In addition, “apoptosis”, “PI3K-Akt signaling pathway”, “MAPK signaling pathway”, and “complement and coagulation cascades” were also enriched in Cluster 2. Genes in Cluster 3 were mainly associated with “wound healing”, “MAPK signaling pathway”, “TNF signaling pathway”, “PI3K-Akt signaling pathway” and “epithelial cell proliferation”. Cluster 5 were mainly involved in “renin-angiotensin system”, “cytokine-cytokine receptor interaction”, “HIF-1 signaling pathway”, “calcium signaling pathway”, “hydrolase activity” (Supplementary Figure S3).

Genes in cluster 7 and cluster 8 showed trapezoidal expression. They mainly enriched in DNA-repair related pathway, such as “DNA replication”, “cell cycle”, “mismatch repair”, “nucleotide excision repair”, and “recombinational repair”. In addition, genes in cluster 8 also involved in “cellular senescence”, “p53 signaling pathway”, “apoptosis - multiple species”, “gap junction”, “tyrosine metabolism” (Supplementary Figure S4).

### 3.6 GSEA enrichment analysis of key genes and pathways in two key time intervals

According to the results of time-dependent gene expression cluster analysis, gene expression changes were most significant in the interval of 0 min–16 min and 26 min to 28 min. Therefore, we further analyzed the GSEA enrichment changes of these two key time intervals in order to find common and characteristic enrichment pathways. GSEA analysis showed that there were many common enrichment pathways in the 16 min–0 min group and 28 min–26 min group, among which DNA-repair related pathways, such as “DNA replication”, “mismatch repair”, and “nucleotide excision repair”, were all up-regulated in both two key time intervals. While metabolism related pathways, such as “propanoate metabolism”, “steroid hormone biosynthesis”, “Valine, leucine and isoleucine degradation”, and “glyoxylate and dicarboxylate metabolism” were all down-regulated in both two key time intervals (Table 1).

**TABLE 1:** The common enrichment pathways in GSEA analysis of genes in 16min–0min group and 28min–26min group.

	16-0 min group			28-26 min group		
GSEA enrichment pathways	NES	p.adjust	Express trend	NES	p.adjust	Express trend
DNA replication	0.766	0.002	up-regulation	0.62	0.004	up-regulation
IL-17 signaling pathway	0.532	0.002	up-regulation	0.417	0.007	up-regulation
p53 signaling pathway	0.555	0.002	up-regulation	0.44	0.007	up-regulation
Mismatch repair	0.718	0.004	up-regulation	0.571	0.022	up-regulation
Nucleotide excision repair	0.607	0.004	up-regulation	0.514	0.009	up-regulation
Valine, leucine and isoleucine degradation	−0.517	0.006	down-regulation	−0.699	0.002	down-regulation
beta-Alanine metabolism	−0.625	0.008	down-regulation	−0.656	0.002	down-regulation
Propanoate metabolism	−0.638	0.008	down-regulation	−0.629	0.004	down-regulation
Complement and coagulation cascades	−0.432	0.011	down-regulation	−0.463	0.005	down-regulation
Peroxisome	−0.448	0.011	down-regulation	−0.614	0.002	down-regulation
Tryptophan metabolism	−0.544	0.013	down-regulation	−0.672	0.002	down-regulation
Pantothenate and CoA biosynthesis	−0.625	0.022	down-regulation	−0.673	0.002	down-regulation
Butanoate metabolism	−0.608	0.023	down-regulation	−0.685	0.002	down-regulation
Vitamin digestion and absorption	−0.614	0.027	down-regulation	−0.61	0.022	down-regulation
Glyoxylate and dicarboxylate metabolism	−0.567	0.034	down-regulation	−0.654	0.002	down-regulation
Phenylalanine metabolism	−0.62	0.04	down-regulation	−0.719	0.002	down-regulation
Proteasome	−0.594	0.004	down-regulation	0.595	0.002	up-regulation
Protein digestion and absorption	0.467	0.006	up-regulation	−0.592	0.002	down-regulation
ECM-receptor interaction	0.456	0.009	up-regulation	−0.435	0.01	down-regulation

GSEA, gene set enrichment analysis; NES, normalized enrichment score; ECM, extra-cellular matrix.

In addition, immune-related pathways, such as “antigen processing and presentation”, “Th1 and Th2 cell differentiation” were downregulated only in 16 min–0 min group (Table 2); “TNF signaling pathway”, “glutathione metabolism”, “gap junction”, “fatty acid biosynthesis”, and “cell cycle” were upregulated only in 16 min–0 min group. In contrary, metabolism-related pathways, such as “glycolysis/Gluconeogenesis”, “tyrosine metabolism”, “glycine, serine and threonine metabolism”, “PPAR signaling pathway”, “citrate cycle”, and “nitrogen metabolism”, and oxidative stress related pathways, such as “oxidative phosphorylation” were downregulated only in 28 min–26 min group (Table 3).

**TABLE 2:** The unique enrichment pathways in GSEA analysis of genes in 16min–0min group.

GSEA enrichment pathways	NES	p.adjust	Express trend
Cell cycle	0.707	0.002	up-regulation
Homologous recombination	0.682	0.002	up-regulation
Fatty acid biosynthesis	0.605	0.046	up-regulation
Gap junction	0.505	0.002	up-regulation
Glutathione metabolism	0.467	0.021	up-regulation
TNF signaling pathway	0.427	0.009	up-regulation
Antigen processing and presentation	−0.792	0.002	down-regulation
ABC transporters	−0.583	0.004	down-regulation
Th1 and Th2 cell differentiation	−0.574	0.002	down-regulation
Cell adhesion molecules	−0.505	0.004	down-regulation
Th17 cell differentiation	−0.495	0.004	down-regulation
Phagosome	−0.474	0.004	down-regulation
Natural killer cell mediated cytotoxicity	−0.458	0.011	down-regulation

GSEA, gene set enrichment analysis; NES, normalized enrichment score.

**TABLE 3:** The unique enrichment pathways in GSEA analysis of genes in 28min–26min group.

GSEA enrichment pathway	NES	p.adjust	Express trend
Nucleocytoplasmic transport	0.443	0.002	up-regulation
C-type lectin receptor signaling pathway	0.442	0.002	up-regulation
Oxidative phosphorylation	−0.502	0.002	down-regulation
PPAR signaling pathway	−0.557	0.002	down-regulation
Retinol metabolism	−0.561	0.002	down-regulation
Glycolysis/Gluconeogenesis	−0.567	0.002	down-regulation
Starch and sucrose metabolism	−0.619	0.002	down-regulation
Tyrosine metabolism	−0.645	0.002	down-regulation
Histidine metabolism	−0.711	0.002	down-regulation
Carbon metabolism	−0.431	0.003	down-regulation
Glycine, serine and threonine metabolism	−0.567	0.004	down-regulation
Citrate cycle (TCA cycle)	−0.64	0.004	down-regulation
Arginine and proline metabolism	−0.526	0.005	down-regulation
Renin-angiotensin system	−0.604	0.005	down-regulation
Nitrogen metabolism	−0.739	0.005	down-regulation
2-Oxocarboxylic acid metabolism	−0.7	0.006	down-regulation
Biosynthesis of amino acids	−0.439	0.025	down-regulation
Arginine biosynthesis	−0.647	0.026	down-regulation
Pyruvate metabolism	−0.491	0.027	down-regulation
Ascorbate and aldarate metabolism	−0.564	0.027	down-regulation
Sulfur metabolism	−0.709	0.027	down-regulation
Fatty acid metabolism	−0.449	0.032	down-regulation
Collecting duct acid secretion	−0.56	0.036	down-regulation
Endocrine and other factor-regulated calcium reabsorption	−0.437	0.038	down-regulation
Fatty acid degradation	−0.46	0.039	down-regulation
Other glycan degradation	−0.602	0.039	down-regulation
Taurine and hypotaurine metabolism	−0.654	0.039	down-regulation
Cysteine and methionine metabolism	−0.456	0.041	down-regulation

GSEA, gene set enrichment analysis; NES, normalized enrichment score; PPAR, peroxisome proliferators-activated receptors; TCA, tricarboxylic acid cycle.

**FIGURE 6:**
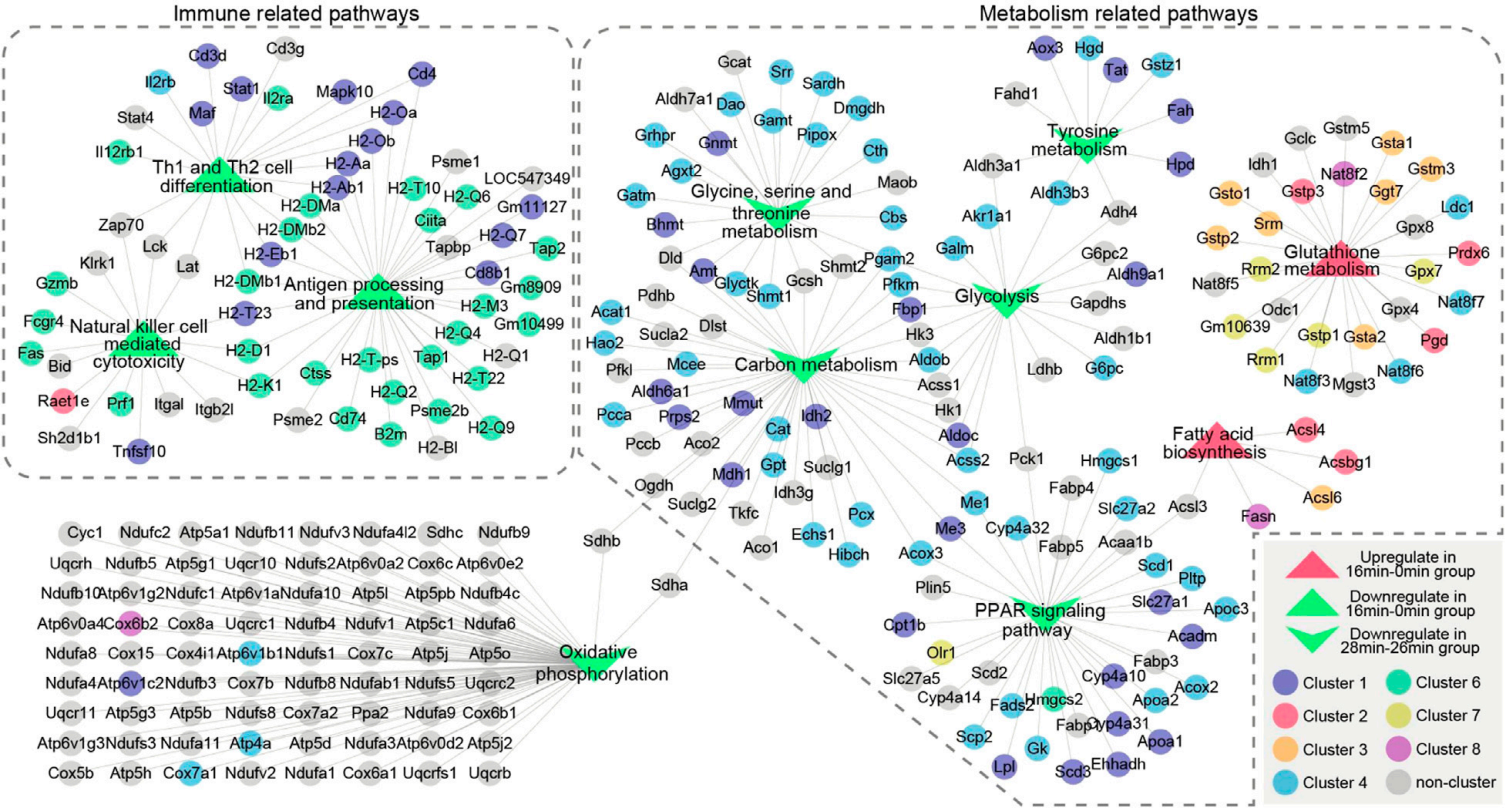
The relationship of unique enrichment pathways in GSEA analysis of genes in 16 min–0 min group or 28 min–26 min group. Two dotted boxes represent functional modules of immune related pathways and metabolism related pathways. The circles represent genes and the triangles represent GSEA enrichment pathways. Different colors represent genes enriched in different clusters. Genes are connected based on the pathways they belong to. GSEA, gene set enrichment analysis.

These results were consistent with previous results of GO and KEGG pathway analysis, namely, gene expression of immune-related pathways was down-regulated in short-term ischemia, while in long-term ischemia, metabolism-related pathways were the mainly enriched pathway. Thus, we performed a more detailed analysis of genes in immune-related pathways and metabolism-related pathways (Figure 6). Genes, such as Pgam2, Idh2, Gnmt, Prdx6, Gsta1, Stat1, and Tnfsf10 were critical genes in maintaining immune and metabolism process.

**FIGURE 7:**
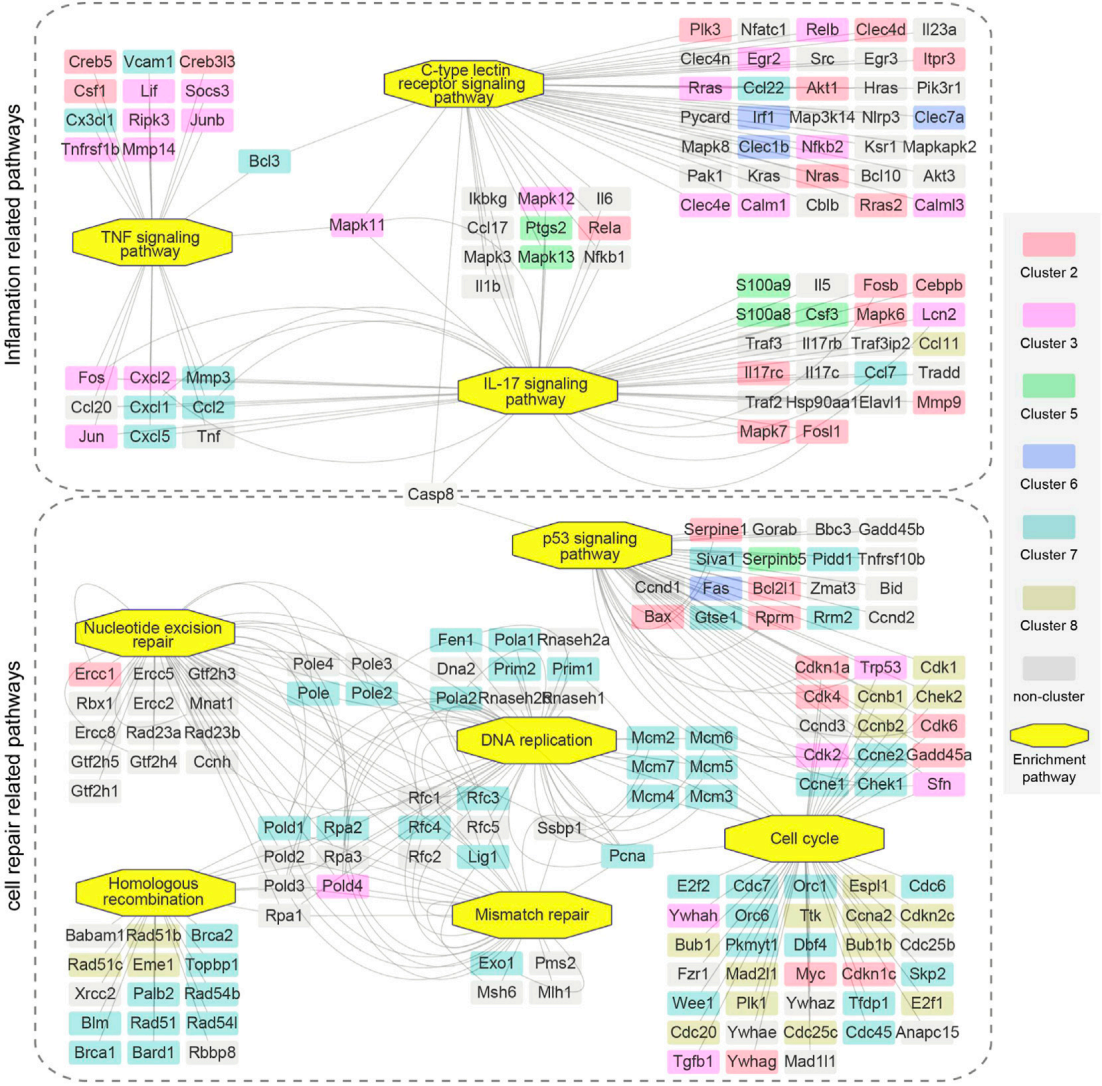
The relationship of upregulated enrichment pathways in GSEA analysis of genes in 16 min–0 min group and 28 min–26 min group. Two dotted boxes represent functional modules of inflamation related pathways and cell repair related pathways. The rectangles represent genes and the octagons represent GSEA enrichment pathways. Different colors represent genes enriched in different clusters. Genes are connected based on the pathways they belong to. GSEA, gene set enrichment analysis.

Furthermore, as indicated above, inflammation related pathways and cell repair related pathways were critical upregulated processes that reflect the kidney injury and repair. Thus, we also performed a more detailed analysis of genes in the pertinent enriched pathways (Figure 7). Pathways in inflammation related pathways and cell repair related pathways were connected by mutually overlapping genes and form an integrated functional module. Mutual genes, such as Fos, Jun, Lcn2, Cxcl1, Cxcl2, Cxcl5, Ccl2, Ptgs2, Mapk11, Mapk12, Bcl3 are critical genes in maintaining inflammation interactions; Trp53, Cdk4, Pold4, Pole2, and Lig1 are critical genes in maintaining cell repair and replications.

### 3.7 Verification of key genes expression

**FIGURE 8:**
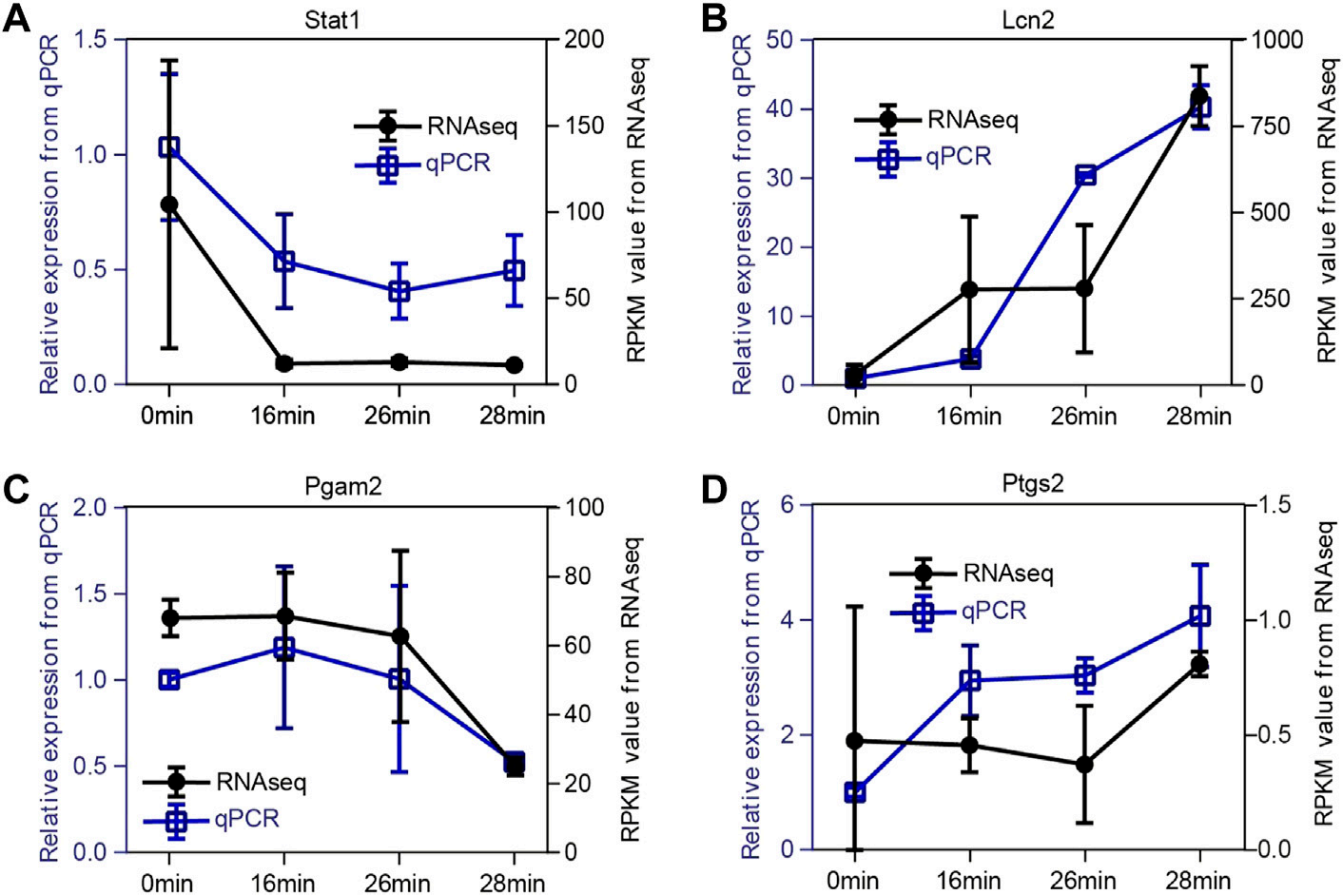
Validation of expression patterns of four representative key genes. **(A–D)** The expression level of Stat1**(A)**, Lcn2**(B)**, Pgam2**(C)**, and Ptgs2**(D)**. To verify the dynamic change of expression level, we selected four representative genes to perform the qPCR assay. The expression patterns of Stat1, Lcn2, Pgam2, and Ptgs2 are highly consistent between RNA-seq data and qPCR results. The x-axis represents the time points. The right black y-axis represents the RPKM value from RNA-seq data, and the left blue y-axis indicates the relative expression level of qPCR results.

In addition, to verify the expression pattern of the key genes, we performed qPCR assays for the four representative genes in the kidney sample (ischemia 0 min, 16 min, 26 min, and 28 min) and compared them with the RNA-seq results. As shown in Figure 8, the expression patterns of Stat1, Lcn2, Pgam2, and Ptgs2 were highly consistent between the RNA-seq and qPCR results. In conclusion, this finding suggests that our RNA-seq data were reliable, and the function and specific mechanism of these key genes in renal injury repair deserve further study.

## 4 Discussion

How many minutes of ischemia time is a safe warm ischemia time of kidney? Different studies have drawn mixed conclusions (Lane et al., 2008; Funahashi et al., 2009; Thompson et al., 2010). What are the pathological changes and molecular characteristics of different renal ischemia time? There are also lacking systematic research.

The experimental mouse models of renal ischemia/reperfusion injury (IRI) can well simulate renal injury caused by renal artery occlusion in human partial nephrectomy. However, there are various surgical methods for IRI model reported in different studies: bilateral IRI (bIRI) (Chen et al., 2018; Chen et al., 2022), contralateral kidney reservation plus unilateral IRI (uIRI) (Godwin et al., 2010; Huang et al., 2022), contralateral nephrectomy plus unilateral IRI (uIRIx) (Ferhat et al., 2018; Wang et al., 2022), unilateral IRI plus contralateral resection 14d before surgery (Chou et al., 2020), and so on (Wei and Dong, 2012; Fu et al., 2018; Wei et al., 2019a; Shiva et al., 2020). The setting of IRI ischemia time varies from 18min to 35min. It has been reported that uIRIx model mice with ischemia greater than 18 min, or bIRI model mice with ischemia greater than 21 min will die within 72 h (Wei et al., 2019a). There were also reports of bilateral ischemia of 21min (Liu et al., 2017), 26 min (Inoue et al., 2016), 28 min (Liu et al., 2014), 30min (Chen et al., 2019), 35 min (Chen et al., 2018), and mice can survive for at least 7 days.

In this study, combined with the experience of previous studies, we established a relatively stable uIRIx model to explore the pathological features, and molecular network characteristics of mouse kidney with different degrees of ischemia. We found that there was no significant difference among 0 min–26 min of ischemia in SCr, while the SCr was dramatically changed between 26 min and 28 min ischemia groups at the condition of 37°C. However, the pathological changes were not exactly the same as SCr. Although there was no change in SCr, renal damage such as cellular casts and renal tubular obstruction could be observed from the group with more than 20 min of ischemia.

Soranno et al. reported that SCr may be poor predictor of long-term histological and functional outcomes after uIRI (Soranno et al., 2019). Slocum et al. also reported that SCr is insensitive, nonspecific, and a late marker of disease (Slocum et al., 2012). Even though 24 h is the peak period of SCr after IRI (Hesketh et al., 2014; Liu et al., 2017), SCr remains unelevated in mild injury, and focusing solely on SCr may mask the severity of renal injury. The SCr at reperfusion 24 h reached the maximum in ischemia 30 min group, and there was no significant difference in SCr between ischemia 60 min and ischemia 30 min (data were not shown) at reperfusion 24 h, which means that SCr also did not totally reflect the extent of acute severe kidney injury.

Some experts reported that both pathological and urea nitrogen (BUN) changes occurred after 18 min of renal ischemia (Kirita et al., 2020), while other reports showed that pathological changes were not significant after 20 min of ischemia (Ide et al., 2021). Such disunity indicates that there are complex influencing factors in the animal model of renal ischemia-reperfusion injury. So far, many factors affect the degree of renal injury after ischemia-reperfusion, including ischemia time, intraoperative kidney temperature, surgical method (unilateral or bilateral renal ischemia-reperfusion), surgical incision site (midline or lateral abdomen), age, gender, and even the type of the nontraumatic microaneurysm clamp, etc. Temperature has a great influence on ischemic tolerance. It has been reported that mice with 2 h of cold ischemia exhibited no significant changes in renal function or histopathology, and 3 h or 4 h of cold ischemia led to mild to moderate acute kidney injury with characteristic features (Wei et al., 2019b). Males are more susceptible to AKI than females, which means female sex protects against development of acute kidney injury (AKI) (Viñas et al., 2020). AKI onset age defines whether the kidney undergoes repair or maladaptive remodeling, the older was less tolerant to injury, and more susceptible to undergo maladaptive repair (Rudman-Melnick et al., 2020).

Other than SCr and BUN, many other injury-related molecules, such as Kim1 (havcr1), Lcn2 (Ngal), and Il6, were also determined to predict kidney damage. Our results showed that the increase of Kim1 and Lcn2 expression emerged at 16 min and 22 min of ischemia respectively, and gradually enhanced according to the ischemia time. However, the expression of Il6 was significantly increased at 30 min of ischemia, with no significant changes in less than 28 min. As can be seen, the molecular expression patterns of each injured molecule are not the same under different ischemic time. Therefore, we further performed high-throughput RNA-Seq.

High-throughput RNA-Seq results also confirmed that the molecular levels of kidney genes had started to change at 16 min–26 min of ischemia whereas SCr showed no difference. The pathology of the 863 DEGs in the 16 min ischemia group presented no alterations. Those results indicate that molecular alterations were faster and earlier than pathology changes and SCr. It is also worth noting whether there will be changes under electron microscopy at the organelle level rather than cellular level. Among the 863 DEGs in the 16 min–0 min group, Kim1 (Havcr1) presented the highest expression DEGs, and the log(fold change) was 4.99. Since Kim1 can be detected in blood and urine (Vinken et al., 2012), and its peak occurred 24 h after IRI (Chen et al., 2022), blood or urine tests for Kim1 may be considered a possible method to detect signs of mild kidney damage”.

For a long time, AKI was considered to be a self-recovery disease, while recent studies have highlighted that AKI can lead to fibrosis and CKD (Venkatachalam et al., 2015; Kormann et al., 2020). When kidney injury is mild and baseline function is normal, the repair process can be adaptive with few long-term consequences. When the injury is more severe, repeated, or to a kidney with underlying disease, the repair can be maladaptive (Canaud and Bonventre, 2015). In order to find the molecular network characteristics of mild to severe ischemia reperfusion kidney injury, and explain key changes in the occurrence of mild and severe injuries, we classified all 4,519 union DEGs identified in high-throughput RNA-Seq into 8 clusters according to the expression patterns.

The expression of genes in Cluster 2 and Cluster 3 were both enhanced with the increase of ischemia degree. Genes in Cluster1, Cluster 4, and Cluster 6 presented reduced expression after ischemia. Inhibiting the function of continuously increased expression genes in Cluster 2 and Cluster 3 or promoting the function of the continuously decreased expression genes in Cluster1 and Cluster 4 may have a potential renal protective effect on AKI. For example, Cdk4 and Cdk6 were members of Cluster 2, Pabla et al. found that G1/S-regulating cyclin dependent kinase 4/6 (CDK4/6) pathway is activated in nephrotoxic AKI (Pabla et al., 2015), and targeted inhibition of CDK4/6 pathway by small-molecule inhibitors resulted in inhibition of cell-cycle progression, amelioration of kidney injury, and improved overall survival.

Targeted inhibition of CDK4/6 pathway by small-molecule inhibitors palbociclib (PD-0332991) and ribociclib (LEE011) resulted in inhibition of cell-cycle progression, amelioration of kidney injury, and improved overall survival (Pabla et al., 2015; Brown et al., 2020; Kim et al., 2020). Kim et al. also reported that mice treated with PD 0332991 before IRI exhibited dramatically reduced epithelial progression through S phase 24 h after IRI and ameliorated kidney injury, as reflected by improved SCr and blood urea nitrogen levels 24 h after injury. The inflammatory markers and macrophage infiltration were also significantly decreased in injured kidneys 3 days following IRI.

The expression of genes in cluster 7 and cluster 8, which mainly enriched in DNA-repair related pathway, increased in mild injury and decreased in severe injury. It was reported that epithelial cell cycle arrest plays an important role in the development of fibrosis (Bonventre, 2014; Lovisa et al., 2015; Moonen et al., 2018). Thus, we speculate that these genes in cluster 7 and cluster 8 may participate in the process of injury repair and play an important role in physiological repair function.

According to the results of time-dependent gene expression cluster analysis, gene expression changes were most significant in two key time intervals, 0 min–16 min and 26 min to 28 min. GSEA enrichment analysis of these two key time intervals were consistence with above results. These results revealed that gene expression of immune-related pathways was down-regulated in short-term ischemia, while in long-term ischemia metabolism-related pathways were the mainly enriched pathway. This finding suggests the important role of metabolic pathways in ischemia-reperfusion.

Recently, single-cell transcriptomic data showed that metabolic processes are critical for maintaining the cellular identity of fully differentiated PT cells, and dysregulation of these pathways underlies the failure of damage-associated PT cells to redifferentiate into normal PT cell state (Ide et al., 2021). Matrix-assisted laser desorption/ionization−mass-spectrometry imaging (MALDI-MSI) results also showed that differential expression of characteristic lipid-degradation products between severe and mild ischemia was detected within 2 h after IRI, even though the histopathological examination revealed no significant difference between kidneys (van Smaalen et al., 2019).

As validated by qPCR assays, the expression patterns of four representative genes, Stat1, Lcn2, Pgam2, and Ptgs2 were highly consistent between the RNA-seq and qPCR results. These key genes were very important in the process of AKI, which could be used to explore some new diagnostic and therapeutic strategies. Signal transducer and activator of transcription 1(STAT1) is a member of STATs, which are important transcription factors that regulate the expression of inflammatory genes (Leonard and O'Shea, 1998). JAK-STAT activation is involved in the pathogenesis of AKI, and is also a key route in the signaling cascade of cytokine mediated AKI (Yun et al., 2021). JAK-STAT inhibitors may prevent and/or treat AKI via blockade of the feedback loop of proinflammatory cytokines, and the inhibition of STAT phosphorylation could also significantly downregulate the production of proinflammatory cytokines during the process of renal inflammation and injury (Li et al., 2019). Lipocalin 2 (LCN2), also known as neutrophil gelatinase associated lipocalin (NGAL), is a secreted protein that belongs to the Lipocalins, and it is also known as an important and common marker of renal injury (Asimakopoulou et al., 2016). Lcn2 is expressed in kidney cells and its production markedly increases in response to stimulation such as ischemia, and plays a critical role in renal ischemia/reperfusion induced AKI by regulating autophagy activation. Glycolytic enzyme phosphoglycerate mutase 2 (Pgam2), is an isoform of Pgam, which converts 3-phosphoglycerate into 2-phosphoglycerate as an isomerase (Mikawa et al., 2021). It was reported that type-M of PGAM2 was specifically expressed in muscles and could be a potential biomarker for early myocardial ischemia (Li et al., 2012), while there is still no related research of Pgam2 in kidney injury. Prostaglandin-endoperoxide synthase 2 (Ptgs2), also called cyclooxygenase-2(Cox-2), is positively correlated with the severity of the inflammatory response (Fan et al., 2021). Ptgs2 was reported upregulated after AKI in our previously research (Chen et al., 2020). Inhibition of Ptgs2 plays as a renal protective role against ischemia-induced damage in AKI (Fan et al., 2021; Liu et al., 2021).

The present study has some limitations and drawbacks. First, because we mainly focused on observing molecular network characteristics and pathological features of mild to severe ischemia reperfusion kidney injury, the main experimental observation time was 1d after AKI. Therefore, we did not monitor the long-term prognosis in mild to severe AKI. Second, this study lacks human kidney tissue sample data, which may result in not full applicability in human. Third, the above results, such as the gene expression level and gene function, should be validated by further mechanism experiments. These issues will be illustrated in our future research.

In summary, our results indicated that there was no absolute safe renal warm ischemia time, and “every minute counts”, which means every minute of ischemia increases kidney damage. Warm ischemia 26 min or above in mice makes severe kidney injury, renal pathology and SCr were significantly changed. Warm ischemia between 18 and 26-min leads to mild kidney injury, with changes in pathology and renal molecular expression, while SCr did not changed. No obvious pathological changes under 16 min warm ischemia were observed, while significant differences were found in molecular expression. The results also showed that SCr could not completely reflect the severity of kidney injury. There are two key time intervals in the process of renal ischemia injury, 0 min–16 min and 26 min to 28 min. Gene expression of immune-related pathways were most significantly down-regulated in short-term ischemia, while metabolism-related pathways were the mainly enriched pathway in long-term ischemia.

Taken together, this study provides novel insights into safe renal artery occlusion time in partial nephrectomy, and is of great value for elucidating molecular network characteristics and pathological features of mild to severe ischemia reperfusion kidney injury, and key genes related to metabolism and immune found in this study also provide potential diagnostic and therapeutic biomarkers for AKI.

## Data Availability

The datasets presented in this study are available in the article and online Supplementary Material. All raw RNA-Seq data discussed in this publication have been deposited in NCBI Gene Expression Omnibus with the following ID: GSE192883 and was available at: https://www.ncbi.nlm.nih.gov/geo/query/acc.cgi?acc=GSE192883.
