# Corrigendum: No safe renal warm ischemia time—The molecular network characteristics and pathological features of mild to severe ischemia reperfusion kidney injury

**DOI:** 10.3389/fmolb.2023.1235738

**Published:** 2023-06-27

**Authors:** Ya-Lei Chen, Huai-Kang Li, Lei Wang, Jian-Wen Chen, Xin Ma

**Affiliations:** ^1^ Department of Critical Care Medicine, Capital Medical University Electric Power Teaching Hospital/State Grid Beijing Electric Power Hospital, Beijing, China; ^2^ Senior Department of Urology, The Third Medical Centre of PLA General Hospital, Beijing, China; ^3^ Department of Nephrology, State Key Laboratory of Kidney Diseases, Beijing Key Laboratory of Kidney Disease Research, First Medical Center of Chinese PLA General Hospital, Nephrology Institute of the Chinese People’s Liberation Army, National Clinical Research Center for Kidney Diseases, Beijing, China

**Keywords:** acute kidney injury, pathological features, ischemia reperfusion kidney injury, molecular network characteristics, mild to severe ischemia reperfusion injury

In the published article, an **author name** was incorrectly written as “Huai-Kan Li”. The correct spelling is “Huai-Kang Li”.

The authors apologize for this error and state that this does not change the scientific conclusions of the article in any way. The original article has been updated.

