# Supplementary material for: No safe renal warm ischemia time—The molecular network characteristics and pathological features of mild to severe ischemia reperfusion kidney injury
Source: Front Mol Biosci. 2022 Nov 16;9:1006917. doi: 10.3389/fmolb.2022.1006917 (PMC9709142; doi:10.3389/fmolb.2022.1006917)
Supplement: Supplementary file 1 [file DataSheet1.doc]

**Supplementary materials list:**

**Supplemental Table S1 | Sequences of oligonucleotide primers used for quantitative real-time PCR (qPCR).**

**Supplementary Fig.S1 | Analysis of Modules in 8 Clusters.**

**Supplementary Fig.S2 | The GO and KEGG pathway enrichment analysis of genes in cluster 1, cluster 4, and cluster 6.**

**Supplementary Fig.S3 | The GO and KEGG pathway enrichment analysis of genes in cluster 2, cluster 3, and cluster 5.**

**Supplementary Fig.S4 | The GO and KEGG pathway enrichment analysis of genes in cluster 7, and cluster 8.**

**Supplemental Table S1. Sequences of oligonucleotide primers used for quantitative real-time PCR (qPCR).**

| Gene name | Forward primer | Reverse primer |
| --- | --- | --- |
| 18s | GTAACCCGTTGAACCCCATT | CCATCCAACGGTAGTAGCG |
| Kim1 | ACATATCGTGGAATCACAACGAC | ACAAGCAGAAGATGGGCATTG |
| Il6 | TGATGGATGCTACCAAACTGG | TCTGGCTTTGTCTTTCTTGTTATC |
| Stat1 | CTGTCATCCCGCAGAGAGAA | GAGCAGAGCTGAAACGACCTA |
| Lcn2 | TTTGTTCCAAGCTCCAGGGC | ACTGGTTGTAGTCCGTGGTG |
| Pgam2 | AGGTGAAGATCTGGAGGCGT | CTGGTCGGACATCCCTTCCA |
| Ptgs2 | AGCCCATTGAACCTGGACTG | ACCCAATCAGCGTTTCTCGT |


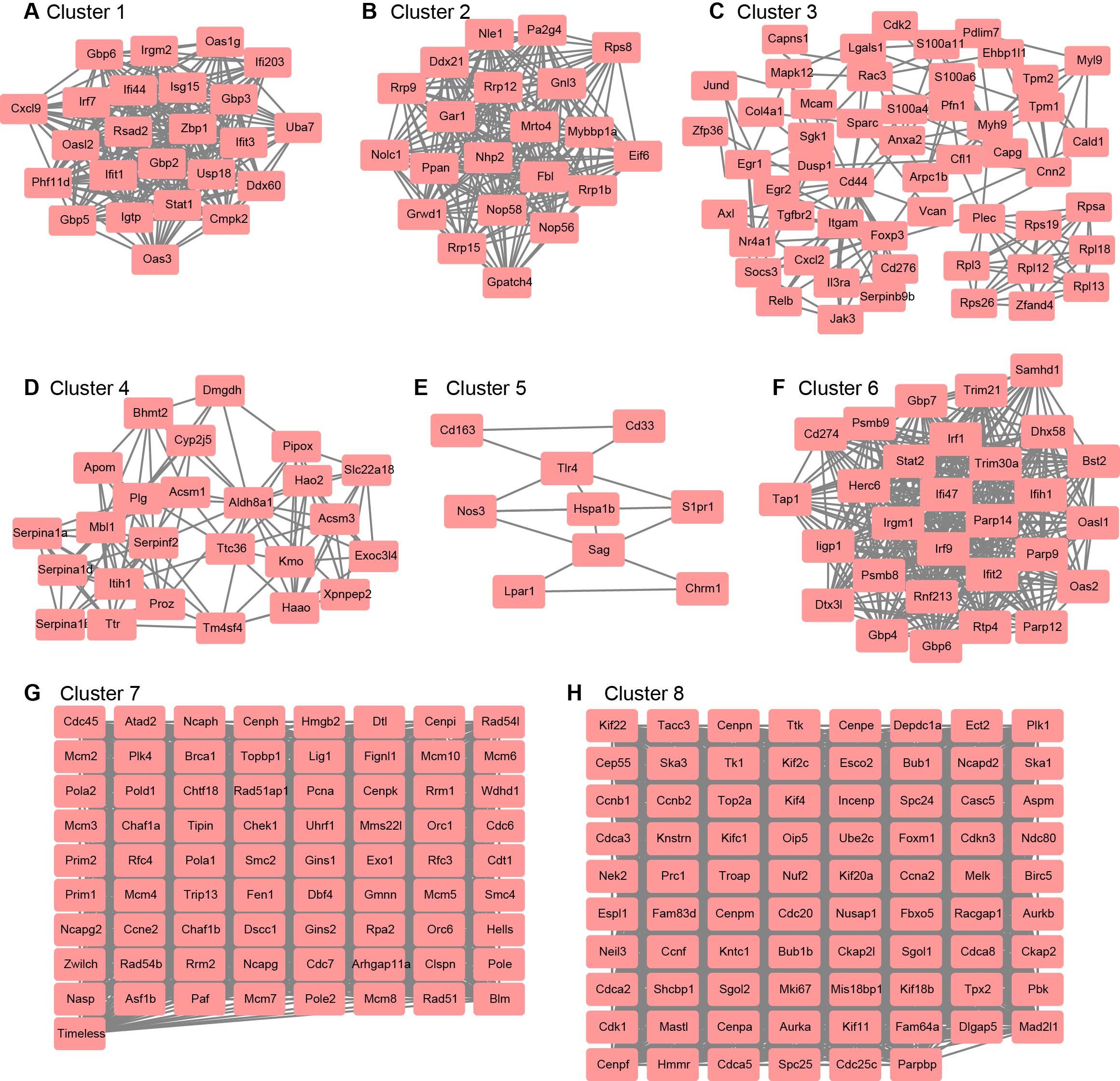


**Supplementary Fig.S1. Analysis of Modules in 8 Clusters.** The first module of Cluster 1(A), Cluster 2(B), Cluster 3(C), Cluster 4(D), Cluster 5(E), Cluster 6(F), Cluster 7(G), and Cluster 8(H) were selected.


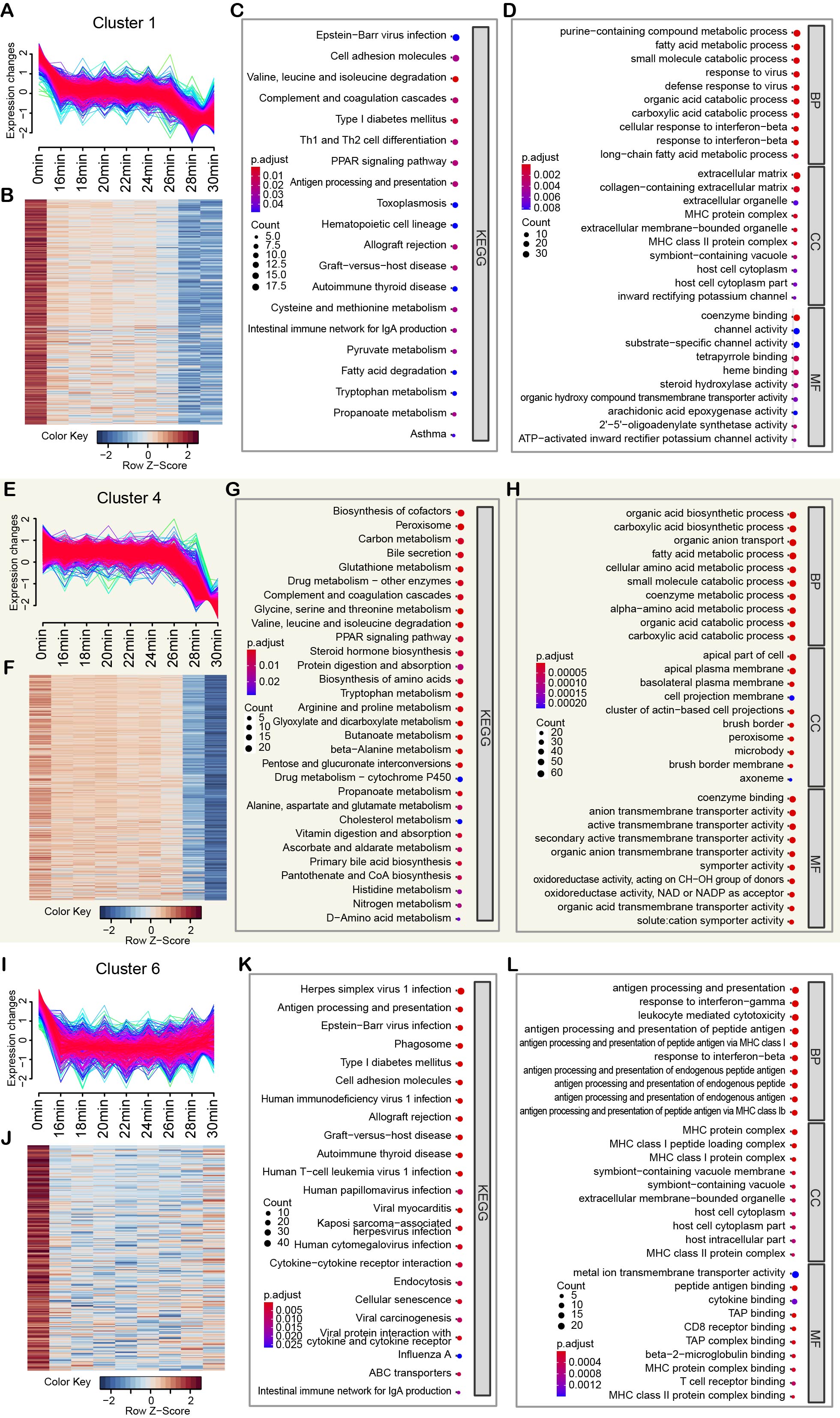


**Supplementary Fig.S2. The GO and KEGG pathway enrichment analysis of genes in cluster 1, cluster 4, and cluster 6.** Gene expressions curve (A), hierarchical cluster analysis heatmap (B), KEGG pathway enrichment analysis bubble diagram(C), and GO enrichment analysis bubble diagram (D) of Cluster 1. Gene expressions curve (E), hierarchical cluster analysis heatmap (F), KEGG pathway enrichment analysis bubble diagram (G), and GO enrichment analysis bubble diagram (H) of Cluster 4. Gene expressions curve (I), hierarchical cluster analysis heatmap (J), KEGG pathway enrichment analysis bubble diagram (K), and GO enrichment analysis bubble diagram (L) of Cluster 6.


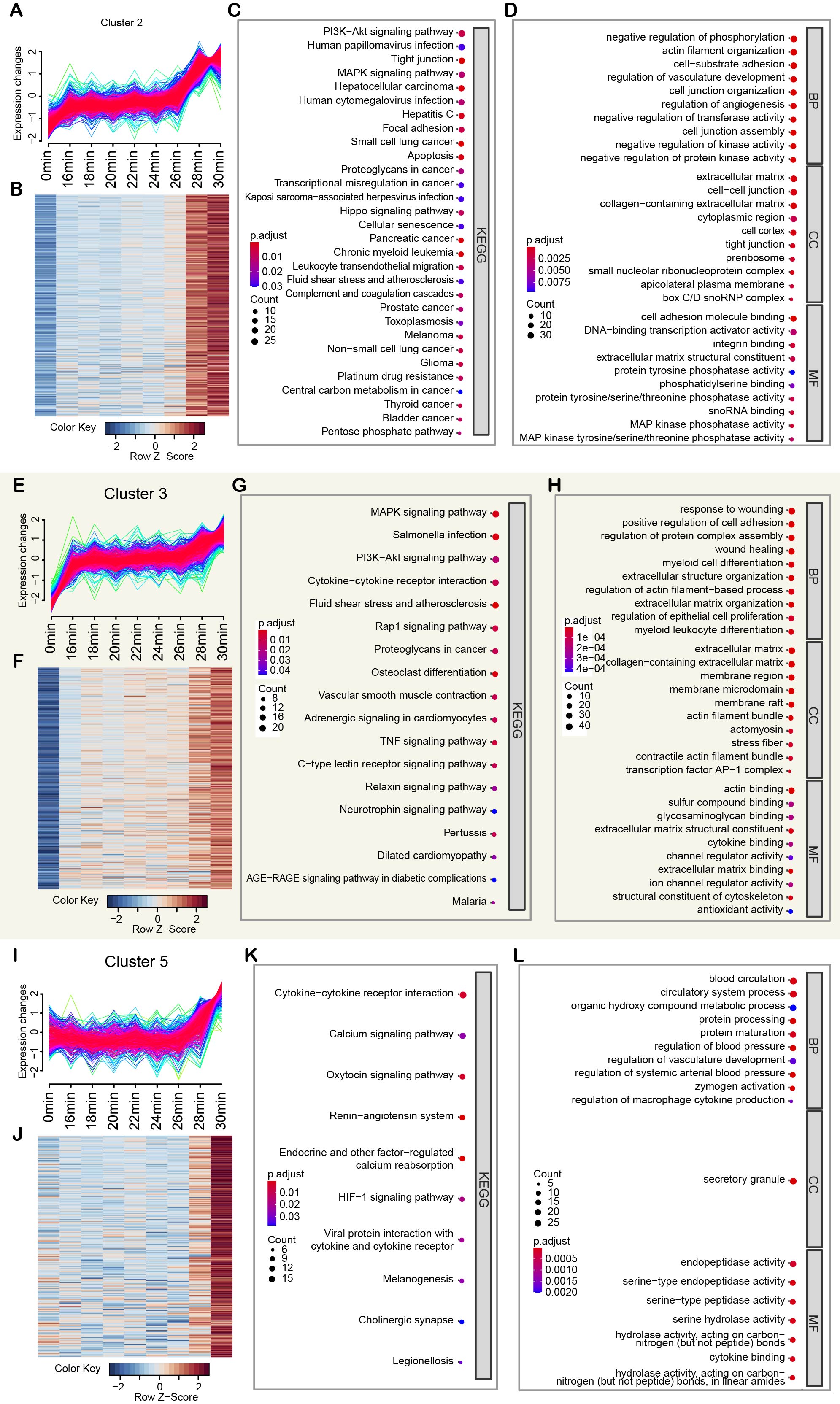


**Supplementary Fig.S3. The GO and KEGG pathway enrichment analysis of genes in cluster 2, cluster 3, and cluster 5.** Gene expressions curve (A), hierarchical cluster analysis heatmap (B), KEGG pathway enrichment analysis bubble diagram(C), and GO enrichment analysis bubble diagram (D) of Cluster 2. Gene expressions curve (E), hierarchical cluster analysis heatmap (F), KEGG pathway enrichment analysis bubble diagram (G), and GO enrichment analysis bubble diagram (H) of Cluster 3. Gene expressions curve (I), hierarchical cluster analysis heatmap (J), KEGG pathway enrichment analysis bubble diagram (K), and GO enrichment analysis bubble diagram (L) of Cluster 5.


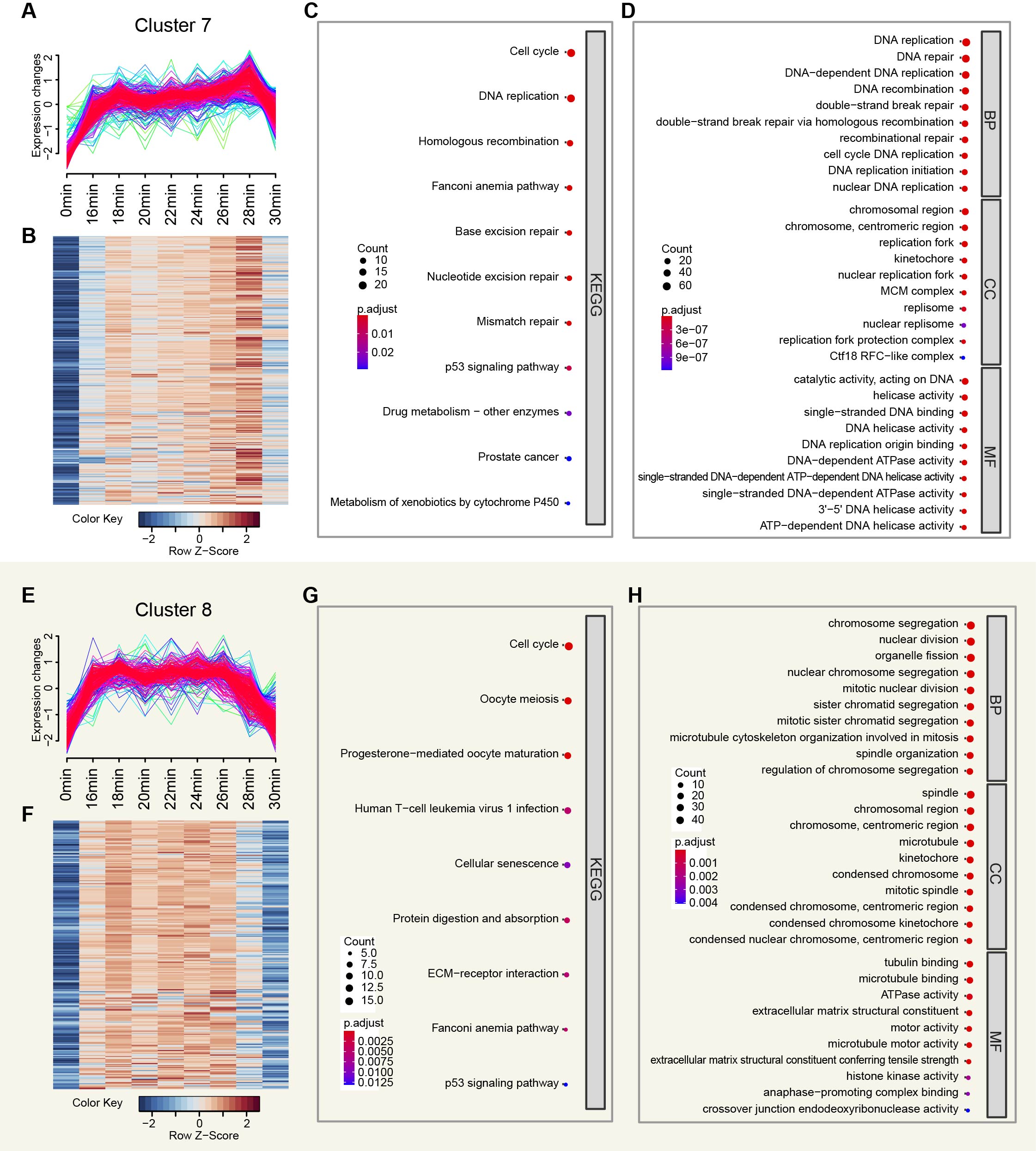


**Supplementary Fig.S****4.** **The GO and KEGG pathway enrichment analysis of genes in cluster 7, and cluster 8.** Gene expressions curve (A), hierarchical cluster analysis heatmap (B), KEGG pathway enrichment analysis bubble diagram(C), and GO enrichment analysis bubble diagram (D) of Cluster 7. Gene expressions curve (E), hierarchical cluster analysis heatmap (F), KEGG pathway enrichment analysis bubble diagram (G), and GO enrichment analysis bubble diagram (H) of Cluster 8.
